# Seroprevalence of anti-*Toxoplasma gondii* antibodies in Egyptian sheep and goats

**DOI:** 10.1186/s12917-018-1440-1

**Published:** 2018-04-02

**Authors:** Yara M. Al-Kappany, Ibrahim E. Abbas, Brecht Devleesschauwer, Pierre Dorny, Malgorzata Jennes, Eric Cox

**Affiliations:** 1 Parasitology Department, Faculty of Veterinary Medicine, Mansoura University, Mansoura, Egypt; 2 Department of Public Health and Surveillance, Scientific Institute of Public Health (WIV-ISP), Rue Juliette Wytsmanstraat 14, 1050 Brussels, Belgium; 3 Laboratory of Parasitology, Faculty of Veterinary Medicine, Ghent University, Merelbeke, Belgium; 4 Department of Biomedical Sciences, Institute of Tropical Medicine, Antwerp, Belgium; 5 Laboratory of Immunology, Faculty of Veterinary Medicine, Ghent University, Merelbeke, Belgium

**Keywords:** Egypt, Filter paper, Small ruminants, *Toxoplasma gondii*, True prevalence

## Abstract

**Background:**

Toxoplasmosis is a zoonotic disease that affects a wide range of animals, including small ruminants. Sheep and goats are considered as biological indicators for the contamination of the environment with *Toxoplasma gondii* oocysts. In addition, in countries such as Egypt, where sheep and goat meat is frequently consumed, *T. gondii* infection in small ruminants may also pose a public health risk. To establish baseline estimates of the prevalence of *T. gondii* infection in Egyptian small ruminants, we used an indirect immunofluorescence assay (IFA) and an enzyme-linked immunosorbent assay (ELISA) to assess the seroprevalence in 398 sheep from four Egyptian governorates (Cairo, Giza, Dakahlia and Sharkia) and in 100 goats from Dakahlia. The positive and negative agreements of both tests were calculated and the true prevalence was estimated using a Bayesian approach.

**Results:**

The true prevalence of antibodies to *T. gondii* as determined by both tests was higher in Egyptian goats (62%) than in sheep for each province (between 4.1 and 26%). Sheep slaughtered at the Cairo abattoir had the lowest true prevalence (4.1%), while true prevalences in Dakahlia, Giza and Sharkia governorates (26%, 23% and 12%, respectively) were substantially higher.

**Conclusions:**

The high prevalence of antibodies to *T. gondii* may indicate an important role of goat and sheep in the transmission of human toxoplasmosis in Egypt, given the habit of eating undercooked grilled mutton.

## Background

Toxoplasmosis is a globally distributed zoonotic disease with important medical and economic implications for man and animals, respectively [1]. Infection of sheep and goats with *Toxoplasma gondii* may cause abortion, stillbirth and neonatal death [2]. *T. gondii* infection in small ruminants also poses a public health risk, since man can acquire *T. gondii* from infected sheep and goats through consumption of undercooked meat, drinking unpasteurized milk or handling raw meat [3].

**Fig. 1:**
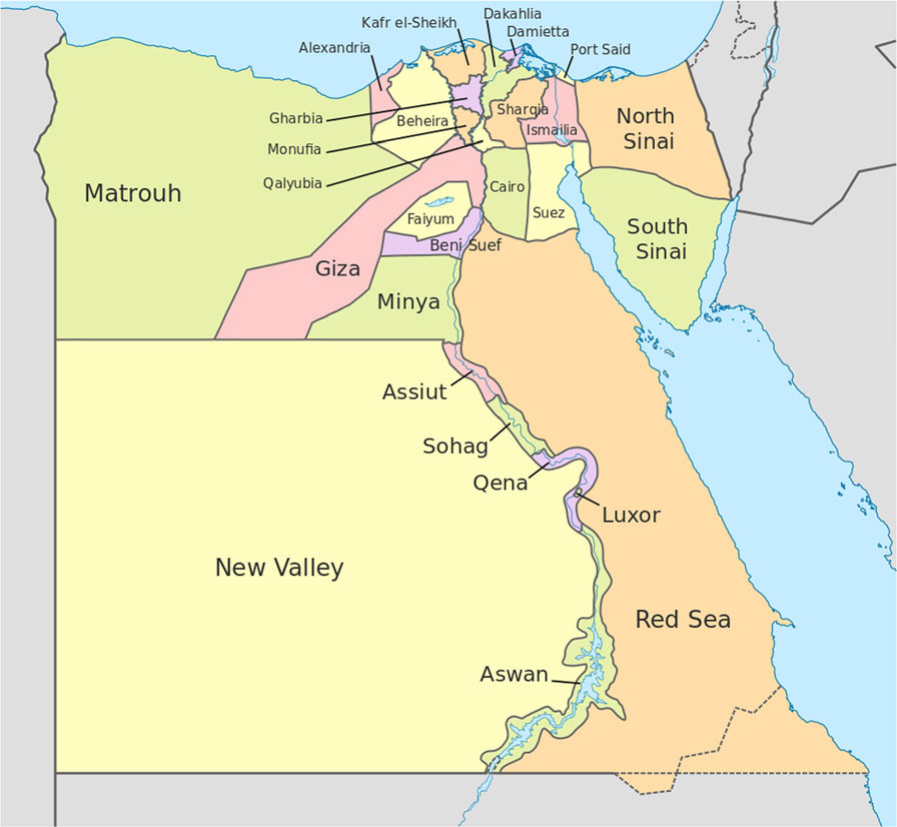
Governorates of Egypt. CC BY-SA 3.0, https://commons.wikimedia.org/w/index.php?curid=32089528

Many studies have assessed the seroprevalence of *T. gondii* in sheep and goats in different parts of the world using different serological techniques [1]. In Egypt, around 10 million small ruminants are yearly produced with slightly more than 50% kept in small herds with less than 10 animals, and 25% owned by people without agricultural land. The prevalence of *T. gondii* infection in sheep in Egypt has been shown to range between 34 and 100% [4, 5], and that in goats between 42 and 60% [5, 6]. However, these studies were limited to one of the Egyptian governorates (respectively, Cairo [4], Faiyum [5], and Giza [6]). Moreover, no previous report investigated the prevalence of *T. gondii* infection in the Dakahlia governorate, one of the Nile delta governorates (Fig. 1).

We aimed to assess the prevalence of anti-*T. gondii* antibodies in sera of Egyptian goats in the Dakahlia governorate and sheep in four Egyptian governorates (Cairo, Dakahlia, Giza and Sharkia).

## Methods

### Sample collection

Blood samples were obtained from 100 goats (reared at villagers’ houses at Dakahlia governorate) and 398 sheep slaughtered at the main abattoir in four Egyptian governorates: Cairo (urban) (*n* = 100), Dakahlia (*n* = 100) and Sharkia (*n* = 99) in the Nile delta and Giza (*n* = 99) (middle Egypt). The Cairo governorate is the most populated of the governorates and completely urbanized. Dakahlia and Sharkia governorates are both located in the Nile Delta, a highly populated agricultural region. The Giza governorate is one of the largest agricultural governorates in Egypt and represents middle Egypt. Sera were collected and stored at − 20 °C until preparation for transport to Belgium and further analysis. Long-term storage of serum normally needs refrigeration or freezing; furthermore, transport of sera from countries with transboundary animal diseases to other countries free of these diseases is not allowed. Therefore, sera were first filtered over a 0.2 μm filter (Pall Life Sciences, USA), thereby removing possible bacterial contaminants [7]. Then, 5 μl from each sample was spotted on Whatman filter paper No 4 (Whatman international Ltd., Maidstone, England), whereafter this paper was heat-treated in a household oven at 60 °C for 180 min to inactivate possible viral contaminants such as foot-and-mouth disease virus and Rift Valley fever virus [8, 9]. An import permit was obtained from the Federal Agency for the Safety of the Food Chain, Belgium (1069371) and the dried filter papers were transported in sealed bags to the Laboratory of Immunology, Faculty of Veterinary Medicine, Ghent University, Belgium. There, each serum spot was cut out the filter paper and dialyzed against 500 μl of phosphate buffered saline (PBS) supplemented with 0.2% Tween®20 (PBS-Tw). Finally, this 1:100 serum dilution in PBS-Tw was used for antibody testing by an indirect immunofluorescence assay (IFA) and an enzyme-linked immunosorbent assay (ELISA).

### Indirect immunofluorescence assay

Formalin-treated tachyzoites of an RH *T. gondii* strain coated on IFA slides (Toxo-Spot® IF, Bio-Mérieux, France) were incubated for 30 min at 37 °C with 50 μl of 1/100 in PBS diluted serum samples. After washing with PBS, the slides were incubated for 30 min at 37 °C with 30 μl of fluorescein isothiocyanate conjugated rabbit-anti-sheep IgG or rabbit-anti-goat IgG (Bethyl Laboratories. Inc., Montgomery USA), diluted in PBS-Evans Blue (counter dye). After washing and drying, the slides were interpreted using a fluorescence microscope. The cut-off read-out of the fluorescence test was established at a dilution of 1/50 with *Toxoplasma* negative and positive sheep reference sera (collected during the 2011 Maedi-Visna screening and validated with the MAT assay), according to the Toxo-Spot® IF guidelines.

### Enzyme-linked immunosorbent assay

All collected sera were tested for the presence of IgG antibodies against *Toxoplasma* total lysate antigen (TLA) according to the ELISA described by Verhelst et al. [10]. Absorbance was read at 405 nm using an iMARK Microplate reader (Biorad, Nazareth, Belgium). The cut-off value, calculated as the mean optical density plus three times the standard deviation of three negative sheep and goat sera (collected during the 2011 Maedi-Visna screening and validated with the MAT assay) assayed at a 1/100 dilution, was 0.395 for sheep and 0.159 for goat.

### Data analysis

Based on the observed test results, we calculated prevalences and corresponding 95% exact confidence intervals (CI) using the prevalence package for R 3.4.0 [11, 12]. Agreement between the results of both serological tests was quantified as the positive agreement index, *PA* = 2*a*/(2*a* + *b* + *c*), and the negative agreement index, *NA* = 2*d*/(2*d* + *b* + *c*), with {*a*, *b*, *c*, *d*} the cell values of the concerned two-by-two table [13]. Confidence intervals for *PA* and *NA* were obtained through bootstrapping using 1,000,000 Dirichlet random deviates, implemented via the mc2d package for R 3.4.0 [12, 14].

As serological assays may yield false positive or false negative results, the observed test results only represent an *apparent* prevalence estimate [15]. To account for the imperfectness of the serological assays and the lack of a gold standard assay, we estimated *true* prevalence in a Bayesian framework, taking into account external information on diagnostic sensitivity and specificity of both assays. We assumed a Beta (1, 1) prior for the true prevalence, and derived prior Beta distributions for the sensitivity and specificity of the IFA and ELISA from Shaapan et al. [4] (Table 1). A Uniform (− 0.25, 0.25) prior was used for the covariance between both tests. Models were implemented independently for the five datasets. For each model, we simulated two chains of 20,000 iterations, of which the first 10,000 were discarded as burn-in. Convergence of the models was visually assessed using trace and density plots and numerically using the multivariate potential scale reduction factor [16]. The models were implemented in R 3.4.0 [12] using the prevalence package version 0.4.0 [11].

**Table 1:** Prior information on diagnostic sensitivity and specificity of the applied assays, based on Shaapan et al. [4]

Test	Sensitivity			Specificity		
	Distribution	Mean	95% UI	Distribution	Mean	95% UI
IFA	Beta(82,20)	0.80	(0.72–0.87)	Beta(181,17)	0.91	(0.87–0.95)
ELISA	Beta(92,10)	0.90	(0.84–0.95)	Beta(170,28)	0.86	(0.81–0.90)

*UI* uncertainty interval, *IFA* indirect immunofluorescence assay, *ELISA* enzyme-linked immunosorbent assay

## Results

### Apparent prevalence and between-test agreement

Table 2 shows the apparent test results per population and per diagnostic test, while Table 3 shows the cross-classification of samples based on both diagnostic tests. Across sheep sera, a positive agreement of 0.78 (95%CI 0.71–0.83) and a negative agreement of 0.92 (0.90–0.94) was found. Across goat sera, a positive agreement of 0.94 (95%CI 0.88–0.97) and a negative agreement of 0.92 (0.85–0.97) was found.

**Table 2:** Positive samples (*x*) and apparent prevalence (*AP*, %) with 95% exact confidence interval (CI) for *Toxoplasma gondii* infection by population and diagnostic test

Diagnostic test	Goats, Dakahlia (*n* = 100)		Sheep, Cairo (*n* = 100)		Sheep, Dakahlia (*n* = 100)		Sheep, Giza (*n* = 99)		Sheep, Sharkia (*n* = 99)	
	x	AP (95%CI)	x	AP (95%CI)	x	AP (95%CI)	x	AP (95%CI)	x	AP (95%CI)
IFA	54	54 (44–64)	20	20 (13–29)	38	38 (28–48)	32	32 (23–42)	34	34 (25–45)
ELISA	59	59 (49–69)	12	12 (6.4–20)	27	27 (19–37)	26	26 (18–36)	17	17 (10–26)

*IFA* indirect immunofluorescence assay, *ELISA* enzyme-linked immunosorbent assay

**Table 3:** Classification of samples according to indirect immunofluorescence assay (IFA) and enzyme-linked immunosorbent assay (ELISA)

Diagnostic test		Goats, Dakahlia	Sheep, Cairo	Sheep, Dakahlia	Sheep, Giza	Sheep, Sharkia
IFA	ELISA					
1	1	80	12	27	24	17
1	0	44	8	11	8	17
0	1	2	0	0	2	0
0	0	272	80	62	65	65

1 = positive; 0 = negative

### True prevalence of anti-toxoplasma gondii antibodies in sheep and goats

The true prevalence of anti-*T. gondii* antibodies in the sera from the Egyptian goats was 62% (95% uncertainty interval [UI] 52–73%). The true prevalence in sheep varied between 4.1% and 26% in the four governorates. Specifically, Cairo had the lowest true prevalence (4.1%; 95%UI 0.2–11%), while higher and more similar prevalences were noted in Dakahlia (26%; 95%UI 16–36%) and Giza (23%; 95%UI 14–33%). An intermediate value was found for Sharkia governorate (12%; 95%UI 3.0–21%).

## Discussion

*T. gondii* is widely prevalent in Egyptian sheep and goats, which may indicate an important role of small ruminants in the transmission of human toxoplasmosis in Egypt, especially when considering the Egyptian people’s habit of eating undercooked grilled mutton and goat meat (*Kabab* and *Kofta*) [17]. The true prevalence was approximately two-fold higher in goats than in sheep in Dakahlia. The higher prevalence in goats was in line with Barakat et al. [6], who found prevalences of 55% and 44% in 306 goats and 320 sheep, respectively, from the Giza governorate; however, Ghoneim et al. [5] found prevalences of 42% and near 100% in 10 goats and 61 sheep from the Faiyum governorate, but this could have been due to the very low sample size. A higher *T. gondii* infection in goats than in sheep may be due to differences in herding practices [18], although this requires further investigation. The observed difference may also be confounded by a different age at slaughter; unfortunately, information on the age of the sampled animals was not available. Nonetheless, this high prevalence underlines the need to give extra attention to goats as a source for infection of humans, which is estimated to be 51% among pregnant women in Egypt [19]. Indeed, in most of the Egyptian rural areas, goat meat and milk are important human food products, which could facilitate the spread of toxoplasmosis to humans.

Our sheep study was carried out over four Egyptian Provinces: Cairo, Sharkia, Dakahlia, and Giza. The latter two governorates showed a nearly similar true prevalence (26%, and 23%, respectively), while an intermediate (12%) and very low value (4.1%) was found for Sharkia and Cairo, respectively. This may be attributed to the fact that Cairo (Capital of Egypt) is an urban area, with a lower cat density, unlike the three other rural governorates where cats are kept by the villagers as natural predators. Previous studies found 44% positive sheep sera in the Giza governorate [6], versus 46% and 42% in the Cairo governorate [4, 20]; thus not suggesting a lower prevalence in the urbanized Cairo governorate than in the rural governorates. However, no comparison with the other rural governorates was performed within the same study. More extensive sampling of sheep over time in different slaughterhouses in Cairo and in parallel in one of the three rural governorates will be needed to confirm our observation.

Our prevalences in sheep were lower than in other studies. This could be due to the specific pre-treatment of the serum samples. Nonetheless, previous studies demonstrated a close relation between results of serological tests on blood and serum in comparison with serum elutes from filter paper [8, 21, 22]; furthermore, we clearly showed that the sample treatment still allowed demonstrating *T. gondii*-specific antibodies. However, filter paper elutes have not been used before for demonstrating antibodies against *T. gondii* in sheep and goats. Whether the treatment decreases antibody concentrations and consequently also sensitivity should therefore be assessed. Nonetheless, since all samples were treated in a similar way in the present study, comparison between samples of different governorates remained possible.

A problem of comparing different seroprevalence studies on *T. gondii* infection remains the wide range of serological assays used [23–25]. In humans the Sabin-Feldman test is considered the gold standard, but is expensive and holds risks for the user because of the use of live tachyzoites [26]; the test is therefore rarely used in sheep [27]. More often, IFA, ELISA, an indirect hemagglutination assay or a modified agglutination test have been used [25]. IFA and ELISA are fast techniques with high sensitivity and specificity, suitable for epidemiological studies [26]. Previous studies applying the TLA-ELISA on sheep serum samples in the Netherlands [28] and in Cairo Egypt [4] reported sensitivities of 97.8% and 90.1%, respectively, and specificities of 96.4% and 85.9%, respectively. Furthermore, Verhelst et al. [25] found a 100% agreement between the Toxo-spot IF® and the TLA-ELISA when applied to Belgian sheep sera. This was not the case in the present study, in which the positive agreement was 78% and 94% for sheep and goat sera, respectively, and the negative agreement was 92% for both sheep and goat sera. Several factors could be responsible for this difference, such as the sampled sheep breeds, the *T. gondii* strains infecting sheep in the field, or the presence of cross-reacting protozoa.

Even though the TLA-ELISA and IFA have been used in several studies, they have not been truly validated for use in sheep and goats. Therefore, in most studies several serological assays were performed and results combined using Bayesian modeling [25, 28]. Nonetheless, there is a high need for validation and standardization of serological assays for seroprevalence studies on *T. gondii* in different species throughout the world. The accurate measurement of the seroprevalence in a comparative way would be an invaluable help to identify risk factors for infections in different regions and at a flock level. Furthermore, it is important to note that serological responses are only indicative of exposure to parasite antigens, but are not a definitive indication that the animals that tested positive are actually infected and capable of transmitting the infection to humans. For any definitive statement on the human health risk to be made samples of meat or milk would need to be tested by mouse bioassay to prove infectivity.

## Conclusion

For the first time, we compared the prevalence of *T. gondii* infection in sheep across different Egyptian governorates, and demonstrated the prevalence of *T. gondii* infection in small ruminants from the Dakahlia governorate, one of the Nile delta governorates. The high prevalence of antibodies to *T. gondii* may indicate an important role of sheep and goat in the transmission of human toxoplasmosis in Egypt. Further validation of serological assays and methods, as well as molecular characterization studies, may provide further evidence on the public health risk of small ruminant toxoplasmosis in Egypt.

## Abbreviations

AP: Apparent prevalence
CI: Confidence interval
ELISA: Enzyme-linked immunosorbent assay
IFA: Indirect immunofluorescence assay
NA: Negative agreement index
PA: Positive agreement index
PBS: Phosphate buffered saline
PBS-Tw: Phosphate buffered saline supplemented with 0.2% Tween®20
TLA: Total lysate antigen
UI: Uncertainty interval
